# Multicenter phase II study of FOLFIRI plus bevacizumab after discontinuation of oxaliplatin-based regimen for advanced or recurrent colorectal cancer (CR0802)

**DOI:** 10.1186/s12885-015-1175-3

**Published:** 2015-03-25

**Authors:** Mitsukuni Suenaga, Tomohiro Nishina, Nobuyuki Mizunuma, Hisateru Yasui, Takashi Ura, Tadamichi Denda, Junichi Ikeda, Taito Esaki, Hogara Nishisaki, Yoshinao Takano, Yasuyuki Sugiyama, Kei Muro

**Affiliations:** 1 Cancer Institute Hospital of the Japanese Foundation for Cancer Research, 3-8-31, Ariake, Koto-ku, Tokyo 135-8550 Japan; 2 National Hospital Organization Shikoku Cancer Center, Kou 160 Minamiumemotomachi, Matsuyama, Ehime 791-0280 Japan; 3 National Hospital Organization Kyoto Medical Center, 1-1, Mukaihata-cho, Fukakusa, Fushimi-ku, Kyoto-shi, Kyoto, 612-8555 Japan; 4 Aichi Cancer Center Hospital, 1-1, Kanokoden, Chikusa-ku, Nagoya, 464-8681 Japan; 5 Chiba Cancer Center, 666-2, Nitona-cho, Chuo-ku, Chiba 260-0801 Japan; 6 Japanese Red Cross Kitami Hospital, North-6, East-2, Kitami, Hokkaido 090-0026 Japan; 7 National Kyushu Cancer Center, 3-1-1, Notame, Minami-ku, Fukuoka, 811-1395 Japan; 8 Hyogo Cancer Center, 13-70, Kitaouji-chou, Akashi, Hyogo 672-8558 Japan; 9 Southern Tohoku General Hospital, 7-115, Yatsuyamada, Koriyama, Fukushima 963-8563 Japan; 10 Teikyo University School of Medicine University Hospital, Mizonokuchi, 3-8-3, Mizonokuchi, Takatsu-ku, Kawasaki, Kanagawa 213-8507 Japan

## Abstract

**Background:**

To investigate the efficacy and safety of FOLFIRI plus bevacizumab regimen with irinotecan (180 mg/m^2^) in patients with advanced or recurrent colorectal cancer who were of the wild-type or heterozygous group for UGT1A1*28 and *6 polymorphisms and discontinued to oxaliplatin-based regimen, prospectively.

**Methods:**

The study population consisted of patients who had discontinued oxaliplatin-based regimen for any reason. The primary endpoint was the response rate. FOLFIRI and bevacizumab regimen [irinotecan: 180 mg/m^2^, 5-fluorouracil infusion: 2400 mg/m^2^, 5-fluorouracil bolus: 400 mg/m^2^, levofolinate calcium: 200 mg/m^2^, bevacizumab: 5 mg/kg] was repeated every 2 weeks for up to 24 cycles.

**Results:**

Ninety-four patients were enrolled; 93 patients were evaluated on safety, 94 patients on efficacy. The response rate was 10.1% (95% confidence interval (CI): 4.7-18.3%). The median time to treatment failure, progression-free survival, and overall survival were 4.1 months (95% CI: 2.8-4.8 months), 5.4 months (95% CI: 4.1-6.2 months), and 14.5 months (95% CI: 11.8-17.0 months), respectively. The treatment-related death was 1.1%, and the early death ≤30 days after the last study treatment was 1.1%. The incidence of grade 3 or higher adverse events was 60.2% for neutropenia, 23.7% for leukopenia, 9.7% for diarrhea, 6.5% for anorexia, and 5.4% for fatigue. All these adverse events and other adverse events were controllable.

**Conclusions:**

FOLFIRI plus bevacizumab regimen with an initial irinotecan dose of 180 mg/m^2^ exhibited an adequate antitumor effect and was confirmed to be manageable and tolerable in Japanese patients with advanced or recurrent colorectal cancer, who had discontinued oxaliplatin-based regimen.

**Trial registration:**

UMIN000001817.

## Background

In 2008, the number of newly diagnosed patients with colorectal cancer exceeded 1,200,000 worldwide, resulting in estimated 608,700 deaths. Colorectal cancer is the third most common cancer among males and the second among females [1]. In Japan, 357,305 people died of cancer in 2011; of them, colorectal cancer accounted for 11.7% among males as the third most common cancer behind lung cancer and gastric cancer, and 14.5% among females as the most common cause of cancer death [2]. Chemotherapy for advanced or recurrent colorectal cancer in Europe and the United States used to be centered on 5-fluorouracil (5-FU) given either as a single agent or in combination with other agents [3]. After the approval of irinotecan and oxaliplatin, however, several clinical trials were reported demonstrating the utility of first-line treatment with FOLFIRI [irinotecan + 5-FU bolus + 5-FU infusion + LV] regimen and FOLFOX [oxaliplatin + 5-FU bolus + 5-FU infusion + LV] regimen [4-6].

In recent years, prolonged overall survival (OS) and progression-free survival (PFS) by the introduction of molecular-targeted agents has been reported [7-9]. Moreover, various studies indicated the possibility that continuation of bevacizumab after first progression (bevacizumab beyond progression; BBP) might contribute to prolonged survival, leading to studies on combined use of molecular-targeted agents [10-12].

However, there have been few reports on FOLFIRI regimen or FOLFIRI plus bevacizumab regimen used in previously-treated patients. Besides, no prospective study using an irinotecan dose of 180 mg/m^2^, the same dose used in FOLFIRI regimen or FOLFIRI plus bevacizumab regimen used in Europe and the United States [5,6], has been reported in Japan.

On the other hand, many genetic researches on UDP glucuronyl transferase (UGT) which is the major metabolic enzyme of an active metabolite of irinotecan, a key drug for chemotherapy of colorectal cancer, have been reported in recent years. Irinotecan was associated with serious adverse events (especially neutropenia) particularly in patients with a variant UGT1A1*28, a polymorphism of UGT [13,14]. Satoh et al. reported that irinotecan could be used more safely in patients of the wild-type or heterozygous group for UGT1A1*28 and *6 polymorphisms because the AUC_0-24h_ of SN-38 was lower and thus the incidences of grade 3 or higher neutropenia and leukopenia were lower in those patients compared with the homozygous group [15].

Consequently, we conducted the first prospective multicenter phase II study in Japan to investigate the efficacy and safety of FOLFIRI plus bevacizumab regimen with the irinotecan dose set to 180 mg/m^2^ in compliance with Good Clinical Practice (GCP) guidelines in patients with advanced or recurrent colorectal cancer who were of the wild-type or heterozygous group for UGT1A1*28 and *6 polymorphisms and had discontinued oxaliplatin-based regimen.

## Methods

Patients who met the following major eligibility criteria were enrolled: aged ≥20 years; Eastern Cooperative Oncology Group performance status (ECOG PS) of ≤ 1; histologically confirmed colorectal cancer; discontinuation for refractory or intolerable to first-line oxaliplatin-based regimen; not previously treated with irinotecan; both UGT1A1*28 and *6 polymorphisms were wild-type (wild-type group) or one of them was heterozygous (heterozygous group); at least one measurable lesion; life expectancy ≥3 months; adequate function of principal organs; and written informed consent. This study was conducted according to a protocol that complied with the Declaration of Helsinki and GCP, and was approved by the Institutional Review Board of each institution as follows: institutional review board of Cancer Institute Hospital of the Japanese Foundation for Cancer Research, institutional review board of National Hospital Organization Shikoku Cancer Center, institutional review board of Aichi Cancer Center Hospital, institutional review board of Chiba Cancer Center, institutional review board of Japanese Red Cross Kitami Hospital, institutional review board of National Kyushu Cancer Center, institutional review board of Hyogo Cancer Center, and institutional review board of Southern Tohoku General Hospital. The study was also registered in the UMIN Clinical Trial Registry as UMIN000001817 (https://upload.umin.ac.jp/cgi-open-bin/ctr/ctr.cgi?function=brows&action=brows&type=summary&recptno=R000002193&language=E).

In this study, FOLFIRI plus bevacizumab regimen [irinotecan (Yakult Honsha Co., Ltd, Tokyo, Japan): 180 mg/m^2^, 5-FU bolus: 400 mg/m^2^, 5-FU infusion: 2400 mg/m^2^, levofolinate calcium: 200 mg/m^2^, bevacizumab: 5 mg/kg] were administered biweekly. The study treatment was defined as FOLFIRI plus bevacizumab and sLV5FU2 with/without bevacizumab after discontinuation of irinotecan, and the treatment was to be continued for up to 24 cycles unless it was discontinued at the discretion of the physician due to progression of the primary disease, onset of adverse events, patient refusal, tumor resection, etc. Even in the patients who had the doses of 5-FU reduced during the first-line treatment, the study treatment was started at the doses of 5-FU described above. In addition, the study treatment was delayed unless all of the following criteria were met on the day of administration or the previous day: leukocyte count ≥ 3,000/mm^3^; platelet count ≥ 100,000/mm^3^; aspartate aminotransferase ≤ 100 IU/L; alanine aminotransferase ≤ 100 IU/L; total bilirubin ≤ 1.5 mg/dL; serum creatinine ≤ 1.50 mg/dL; protein urine, bleeding, stomatitis and fatigue grade 1≥; and no diarrhea.

The irinotecan dose was reduced to 150 mg/m^2^, 125 mg/m^2^ or 100 mg/m^2^ if grade 4 neutropenia or grade 3 or higher thrombocytopenia or diarrhea occurred. As for the doses of 5-FU, the infusion dose was reduced to 2,000 mg/m^2^ or 1,600 mg/m^2^ and the bolus dose to 200 mg/m^2^ when grade 4 neutropenia or grade 3 or higher thrombocytopenia, diarrhea, stomatitis or hand-foot syndrome occurred. No post chemotherapy was specified in this study.

The dose intensity (DI) was calculated as the ratio of the total dose (expressed in milligrams) per square meter divided by total treatment duration. The relative DI (RDI) was calculated as the ratio of the DI actually delivered to that of the DI proposed in the protocol.

The primary endpoint was response rate, and the secondary endpoints were time to treatment failure (TTF), PFS, OS, dose intensity, and incidence of adverse events. Tumor response was evaluated according to the RECIST (ver. 1.0) by independent extramural review board. Adverse events were evaluated according to the NCI-CTC (ver. 3.0).

Duration of stable disease, TTF, PFS and OS were calculated using the Kaplan-Meier method. Duration of stable disease was defined as the period from the date of the first dose to the date of the first imaging showing progressive disease by the non-institutional review. TTF was defined as the time from the date of the first dose to the date of treatment discontinuation, or the date of progression, or the date of death from any cause, whichever was earlier. Data on patients who had completed 24 cycles of treatment or who discontinued treatment for tumor resection were censored at the date of final observation. PFS was defined as the time from the date of the first dose to the date of progression or the date of death from any cause, whichever was earlier. Patients undergoing resection of metastases were censored at the time of surgery. OS was defined as the time from the date of the first dose of the study treatment to the date of death from any cause.

In the V308 study, the response rate of FOLFIRI regimen (CPT-11 180 mg/m^2^) in FOLFOX-received patients who discontinued the treatment due to progression of the primary disease or adverse event was reported to be 4%. Also, the E3200 study reported that the response rate was 8.6% in the FOLFOX group and 22.7% in the FOLFOX plus bevacizumab group. Based on these results, the expected response rate of FOLFIRI plus bevacizumab regimen was set to be 12% in this study [6,16]. At this rate, 84 patients would be required to make the width of 95% confidence interval (CI) of the response rate about 15%, and therefore 90 patients were set to take into account those who would be excluded from the analysis set.

The chi-square test for independence (Fisher’s exact test when the expected value was <5) was performed to compare the response rate in patients who received first-line chemotherapy with/without bevacizumab. To compare PFS and OS of the first-line treatment with/without bevacizumab, point estimates of the hazard ratio (HR) were made by Cox regression analysis (proportional hazards model) and the log-rank test was performed. The chi-square test for independence (Fisher’s exact test when the expected value was <5) was employed to compare the incidence of adverse events for *UGT1A1* polymorphisms.

## Results

### Patient characteristics

Between May 2009 and November 2010, 94 patients from 18 institutions were registered. Of these, 1 patient was withdrawn before starting the study treatment, 1 patient was found to be not eligible after enrollment, 1 patient became a candidate for surgery after 1 cycle of treatment, and 2 patients were found to have no target lesion by the independent extramural review board before enrollment in this study. Consequently, 93 patients, excluding the patient who did not receive the study treatment, were included in the safety analysis set, and 89 patients were included in the efficacy analysis set. Details of patient characteristics are shown in Table 1. There was no patient who treated with anti-EGFR agents as a first-line chemotherapy.

**Table 1:** **Patient characteristics at baseline (N = 89)**

Characteristic	N	%
Sex		
Male	49	55.1
Female	40	44.9
Age		
Median	62	
Range	36-80	
ECOG PS		
0	66	74.2
1	23	25.8
Primary tumor		
Colon	56	62.9
Rectum	33	37.1
Histological classification		
Tubular adenocarcinoma (well-differentiated type)	27	30.3
Tubular adenocarcinoma (moderately-differentiated type)	50	56.2
Poorly-differentiated adenocarcinoma	9	10.1
Mucinous adenocarcinoma	3	3.4
Number of metastatic sites		
1	37	41.6
2≤	52	58.4
UGT1A1*28/*6 polymorphism		
Wild-type group	52	58.4
Heterozygous group	37	41.6
First-line treatment		
Refractory (PD) / intolerable (adverse event) to oxaliplatin-based treatment	46/43	51.7/48.3
With/without bevacizumab	69/20	77.5/22.5

### Treatment

The median cycles of the study treatment was 8.0 (range: 1–24), and 7 patients (7.9%) were able to continue FOLFIRI plus bevacizumab throughout the 24 cycles. The median RDI of each agent was 80.0% for irinotecan, 75.7% for 5-FU bolus, 80.0% for 5-FU infusion, and 80.0% for bevacizumab. Dose modification of each agent was as shown in Table 2. The major reasons for reducing the irinotecan dose were neutropenia in 10 patients (11.2%) and diarrhea in 6 patients (6.7%), and those for 5-FU were neutropenia in 12 patients (13.5%), diarrhea in 2 patients (2.2%), and stomatitis in 2 patients (2.2%). There were 26 patients (29.2%) who had the dose of first-line 5-FU reduced. Of these, 17 patients (19.1%) also had the 5-FU dose reduced in this study, and 9 patients (10.1%) didn’t. Four patients (4.5%) had the doses of both irinotecan and 5-FU reduced simultaneously due to diarrhea.

**Table 2:** **Dose modification of Irinotecan and 5-fluorouracil (5-FU) (N = 89)**

	Dose Level	Dose (mg/m^2^)	N	%	Duration until modification (days)	
					Median	Range
Irinotecan	0	180	52	58.4	-	-
	−1	150	37	41.6	33.0	(14–448)
	−2	125	12	13.5	76.5	(14–146)
	−3	100	3	3.4	112.0	(47–181)
5-FU (bolus/infusion)*	0	400/2,400	55	61.8	-	-
	−1	400/2,000	34	38.2	47.5	(14–448)
	−2	0/1,600	9	10.1	82.0	(33–301)

*No patient had the dose of only bolus or infusion of 5-FU reduced.

The reasons for discontinuing the study treatment included progression of the primary disease in 48 patients (51.6%), adverse events in 24 patients (25.8%), patient refusal in 6 patients (6.5%), and discretion of the physician in 9 patients (9.7%).

### Efficacy

**Figure 1:**
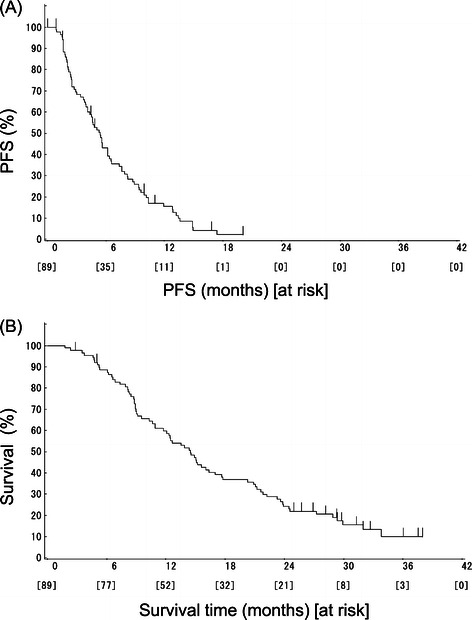
**Kaplan-Meier curves for the study end points. (A)** Progression-free survival (PFS), **(B)** Overall survival (OS).

The results of the efficacy analysis are shown in Table 3. The response rate was 10.1% (95% CI: 4.7-18.3%), and the disease control rate was 65.2% (95% CI: 54.3-75.0%). There was no patient whose best overall response was rated as complete response. The duration of stable disease was 5.4 months (95% CI: 4.1-6.2 months). The median TTF was 4.1 months (95% CI: 2.8-4.8 months), whereas the median PFS was 5.4 months (95% CI: 4.1-6.2 months) (Figure 1A). The median OS was 14.5 months (95% CI: 11.8-17.0 months) (Figure 1B). No difference was noted in efficacy between different groups of *UGT1A1* polymorphisms.

**Table 3:** **Analysis of efficacy (N = 89)**

Endpoint		95% CI
Response rate	10.1%	4.7-18.3%
Disease control rate	65.2%	54.3-75.0%
TTF	4.1 months	2.8-4.8 months
PFS	5.4 months	4.1-6.2 months
OS	14.5 months	11.8-17.0 months

**Figure 2:**
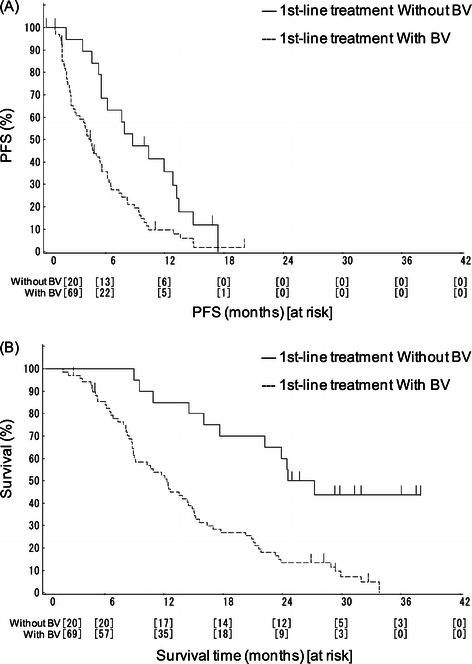
**Kaplan-Meier curves according to first-line treatment (oxaliplatin-based regimen) with/without bevacizumab. (A)** Progresion-free survival (PFS), **(B)** Overall survival (OS).

The response rate, PFS, and OS in patients previously treated with a regimen with bevacizumab were 7.2% (95% CI: 2.4-16.1%), 4.5 months (95% CI: 3.0-5.6 months), and 12.3 months (95% CI: 8.9-14.9 months), respectively, and those in patients previously treated with a regimen without bevacizumab were 20.0% (95% CI: 5.7-43.7%, p = 0.1102), 8.8 months (95% CI: 6.0-13.2 months, HR 2.100, p = 0.0062) and 25.9 months (95% CI: 17.7-NA months, HR 3.650, p < 0.0001) (Figure 2). Additional multivariate analysis including sex, age, ECOG PS, primary tumor site, and pre-treatment with bevacizumab as covariates resulted in HR for OS of 3.773 (p < 0.001) and for PFS of 2.237 (p = 0.008).

The outcome was compared between patients who discontinued the first-line oxaliplatin-based treatment due to progression of the primary disease and those who discontinued it due to adverse events: the response rate, PFS, and OS were 8.7% (95% CI: 2.4-20.8%), 3.8 months (95% CI: 2.5-4.7 months) and 12.4 months (95% CI: 8.9-15.1 months), respectively, in the former, while they were 11.6% (95% CI: 3.9-25.1%), 6.6 months (95% CI: 5.6-9.4 months) and 17.0 months (95% CI: 12.7-23.3 months), respectively, in the latter.

### Toxicity

Table 4 lists the adverse events observed in this study. There was 1 patient (1.1%) of treatment-related death and 1 patient (1.1%) of early death; that is, within 30 days from the last dose. Granulocyte colony-stimulating factor was used in 24 (25.8%) patients.

**Table 4:** **Incidence of adverse events (N = 93)**

Adverse event	≥Grade 1		≥Grade 3	
	N	%	N	%
Neutropenia	89	95.7	56	60.2
Leukopenia	89	95.7	22	23.7
Thrombocytopenia	12	12.9	0	0
Anemia	44	47.3	7	7.5
AST	27	29.0	0	0
ALT	32	34.4	1	1.1
Anorexia	60	64.5	6	6.5
Diarrhea	59	63.4	9	9.7
Nausea	61	65.6	0	0
Stomatitis	62	66.7	3	3.2
Vomiting	36	38.7	0	0
Fatigue	66	71.0	5	5.4
Cholinergic syndrome	18	19.4	0	0
Febrile neutropenia	1	1.1	1	1.1
Sepsis	2	2.2	2	2.2
Interstitial pneumonia	1	1.1	0	0
Hypertension	10	10.8	1	1.1
Bleeding	7	7.5	0	0
Pulmonary artery thrombosis	1	1.1	1	1.1
Pulmonary embolism	1	1.1	0	0
Embolism venous	1	1.1	1	1.1
Gastrointestinal perforation	2	2.2	2	2.2

The major grade 3 or higher adverse events observed in the wild-type group were neutropenia in 33 patients (62.3%), leucopenia in 11 patients (20.8%), diarrhea in 5 patients (9.4%), anorexia in 4 patients (7.5%), and fatigue in 4 patients (7.5%), while the major grade 3 or higher adverse events in the heterozygous group were neutropenia in 23 patients (57.5%, p = 0.6421), leucopenia in 11 patients (27.5%, p = 0.4486), diarrhea in 4 patients (10.0%, p = 0.3014), anorexia in 2 patients (5.0%, p = 0.6964), and fatigue in 1 patient (2.5%, p = 0.3865). No statistically significant difference was observed in any of these adverse events between the wild-type group and the heterozygous group.

### Post chemotherapy

Table 5 shows the post chemotherapy after completion of this study. Of the 24 patients (25.8%) who discontinued the study treatment due to adverse events, 10 patients (10.8%) did not meet the criteria for administration for 15 days or longer after the second cycle, and 5 of them (5.4%) continued FOLFIRI plus bevacizumab or FOLFIRI after recovering from the adverse events. There were 16 patients (18.0%) who received oxaliplatin-based regimen after the study was completed, including 8 patients (9.0%) who were refractory to and 8 patients (9.0%) who were intolerable to oxaliplatin-based regimen.

**Table 5:** **Post chemotherapy treatment (N = 89) Table legend text**

Post chemotherapy	N	%
Irinotecan-based regimen	54	60.7
Oxaliplatin-based regimen	16	18.0
Chemotherapy with bevacizumab	37	41.6
Chemotherapy with cetuximab	32	36.0
Chemotherapy with panitumumab	21	23.6

## Discussion

The BEVACOLOR study on the efficacy and safety of standard chemotherapies such as second-line FOLFIRI or FOLFOX combined with bevacizumab for advanced or recurrent colorectal cancer, and the AVASIRI study on the efficacy and safety of FOLFIRI plus bevacizumab with an initial irinotecan dose of 150 mg/m^2^ have been reported [17,18]. In this study, we demonstrated the efficacy and safety of FOLFIRI plus bevacizumab regimen with an initial irinotecan dose of 180 mg/m^2^ in patients with advanced or recurrent colorectal cancer who discontinued first-line oxaliplatin-based regimen and who were of the wild-type or heterozygous group for *UGT1A1* polymorphisms. The response rate, median PFS, and median OS in this study were 10.1%, 5.4 months, and 14.5 months, respectively. In the recently reported ML18147 study, the response rate, median PFS, and median OS of the group receiving second-line FOLFIRI plus bevacizumab were 5%, 5.7 months, and 11.2 months, respectively, and our results compared favorably with those results [11].

When the patients received the first-line treatment, bevacizumab was not yet approved in Japan. For this reason, 22.5% of the patients who received the study treatment did not use bevacizumab during the first-line treatment. We therefore conducted exploratory analyses to investigate the efficacy of the first-line treatment with/without bevacizumab, and there were statistically significant differences in the median PFS and OS between the patients who received first-line bevacizumab and those who did not (PFS; p = 0.0062, OS; p < 0.001). There was no substantial difference in the patient characteristics and contents of post chemotherapy between the two groups. These results support the results reported by Giantonio et al. in that second-line chemotherapy with bevacizumab is highly effective in patients who do not receive first-line chemotherapy with bevacizumab [16]. However, since the study population included only 20 patients who were not treated with bevacizumab as a first-line, further studies are required to confirm the effective use of bevacizumab. In addition, *KRAS* mutation status on the efficacy was not collected in this study, although frequently reported in the world these days [19-21]. It is because *KRAS* assay was not yet approved in Japan (approved in April, 2010) when our study was started in May 2009, so that patients enrolled in this study did not necessarily examine *KRAS* mutation.

The incidence of adverse events and the median RDIs indicated that the study treatment was tolerated. No new issue of concern was found when compared with previously reported studies [16-18]. In this study, 4 patients (4.5%) had the doses of both irinotecan and 5-FU reduced simultaneously due to diarrhea. In other patients, the agents were safely administered by reducing the dose of either irinotecan or 5-FU. Furthermore, none of the patients in this study had irinotecan or 5-FU dose reduced due to grade 3 or higher diarrhea and grade 3 or higher neutropenia occurring simultaneously or due to severe fatigue. These results further confirmed that FOLFIRI plus bevacizumab with an initial irinotecan dose of 180 mg/m^2^ was a controllable regimen for Japanese patients.

In this study, 10.8% of the treated patients did not recover from adverse events within 15 days and thus discontinued the study treatment. However, half of them (5.4%) were able to continue on FOLFIRI plus bevacizumab or FOLFIRI as post chemotherapy after recovering from the adverse events. In addition, 18.0% of the treated patients received post chemotherapy with oxaliplatin after completing the study, of which 9.0% were those who were refractory to oxaliplatin-based regimen and the remaining 9.0% were those who were intolerable to the regimen. The median survival time among these patients (28.9 months) was longer than the patients who did not receive post chemotherapy with oxaliplatin (12.6 months). Thus, it seems meaningful to consider reintroduction of oxaliplatin in patients who discontinued oxaliplatin-based regimen as a first-line treatment due to severe peripheral neuropathy, provided that it has improved. These results suggest that, even during third-line or later chemotherapy, administration of irinotecan or oxaliplatin, the key drugs for colorectal cancer chemotherapy, would contribute to prolonged survival. More than one clinical trial has been conducted on the reintroduction of oxaliplatin, which reported favorable results [22,23].

This study also included an exploratory assessment of the efficacy and safety of each polymorphism in patients of the wild-type or heterozygous group for UGT1A1*28 and *6 polymorphisms. As a result, no significant difference in efficacy and safety was suggested between these two groups. We consider it necessary in future to determine appropriate doses irinotecan and evaluate the efficacy and safety in Japanese patients of the homozygous group for UGT1A1*28 and *6 polymorphisms.

## Conclusions

FOLFIRI plus bevacizumab regimen with an initial irinotecan dose of 180 mg/m^2^ exhibited an adequate antitumor effect and was confirmed to be manageable and tolerable in Japanese patients with advanced or recurrent colorectal cancer who had discontinued oxaliplatin-based regimen.

## Abbreviations

5-FU: 5-fluorouracil
BBP: Bevacizumab beyond progression
CI: Confidence interval
DI: Dose intensity
ECOG PS: Eastern cooperative oncology group performance status
GCP: Good clinical practice
HR: Hazard ratio
N: Number of patients
OS: Overall survival
PFS: Progression-free survival
RDI: Relative dose intensity
TTF: Time to treatment failure
UGT: UDP glucuronyl transferase
